# Bias due to differential and non-differential disease- and exposure misclassification in studies of vaccine effectiveness

**DOI:** 10.1371/journal.pone.0199180

**Published:** 2018-06-15

**Authors:** Tom De Smedt, Elizabeth Merrall, Denis Macina, Silvia Perez-Vilar, Nick Andrews, Kaatje Bollaerts

**Affiliations:** 1 P95 Epidemiology and Pharmacovigilance, Leuven, Belgium; 2 GSK Vaccines, Amsterdam, The Netherlands; 3 Sanofi Pasteur, Lyon, France; 4 FISABIO-Public Health, Valencia, Spain; 5 Erasmus University Medical Center, Rotterdam, The Netherlands; 6 Statistics, Modelling, and Economics Department, Public Health England, Colindale, London, United Kingdom; Universidad Nacional de la Plata, ARGENTINA

## Abstract

**Background:**

Studies of vaccine effectiveness (VE) rely on accurate identification of vaccination and cases of vaccine-preventable disease. In practice, diagnostic tests, clinical case definitions and vaccination records often present inaccuracies, leading to biased VE estimates. Previous studies investigated the impact of non-differential disease misclassification on VE estimation.

**Methods:**

We explored, through simulation, the impact of non-differential and differential disease- and exposure misclassification when estimating VE using cohort, case-control, test-negative case-control and case-cohort designs. The impact of misclassification on the estimated VE is demonstrated for VE studies on childhood seasonal influenza and pertussis vaccination. We additionally developed a web-application graphically presenting bias for user-selected parameters.

**Results:**

Depending on the scenario, the misclassification parameters had differing impacts. Decreased exposure specificity had greatest impact for influenza VE estimation when vaccination coverage was low. Decreased exposure sensitivity had greatest impact for pertussis VE estimation for which high vaccination coverage is typically achieved. The impact of the exposure misclassification parameters was found to be more noticeable than that of the disease misclassification parameters. When misclassification is limited, all study designs perform equally. In case of substantial (differential) disease misclassification, the test-negative design performs worse.

**Conclusions:**

Misclassification can lead to significant bias in VE estimates and its impact strongly depends on the scenario. We developed a web-application for assessing the potential (joint) impact of possibly differential disease- and exposure misclassification that can be modified by users to their own study scenario. Our results and the simulation tool may be used to guide better design, conduct and interpretation of future VE studies.

## Introduction

Vaccine effectiveness (VE) is defined as a measure of protection among vaccinated persons attributable to a vaccine administered under field conditions to a given population, which is different from vaccine efficacy being defined as the effect of vaccination among vaccinated persons as measured in pre-licensure clinical trials with vaccination allocated under optimal conditions [1]. Whilst aggregated data may be used for assessment of impact and uptake, individual level data are usually required to estimate VE. Such data may be available nationally, regionally or in health systems which are nationally representative.

When studying VE, it is essential to accurately identify cases of the vaccine preventable disease and the vaccination status (e.g. defined as 1 dose vs none, 2 doses vs 1 dose, or completely vs partially vaccinated, depending on the research question of interest). Indeed, assuming misclassification is non-differential and independent of other errors, both disease and exposure misclassifications tend to bias the VE estimates toward the null [2]. Disease and exposure statuses may reciprocally affect each other’s ascertainment (i.e. differential misclassification) and lead to biased estimates in either direction [3]. For example, differential disease misclassification might arise from differences in healthcare seeking behavior, with subjects more likely to seek care being more likely vaccinated and also being more likely correctly diagnosed as diseased. Laboratory confirmation is desirable when assessing VE [4]. However, laboratory test results are not always available or perfectly accurate and, especially in health care database-based analyses, case definitions often rely on clinical criteria, potentially resulting in disease misclassification. Different sources of disease misclassification exist and they might be broadly categorized as under-ascertainment (individuals that do not seek healthcare) and underreporting (individuals that do seek healthcare, but whose health event is not accurately captured due to various reasons) [5]. Likewise, the vaccination exposure information might be subject to coding entry error or omissions potentially biasing estimates of VE as well [6].

Concerns regarding disease and exposure misclassifications are particularly relevant when conducting epidemiological studies using health care databases [7]. Nonetheless, and despite concerns on data validity, sample representativity and the limited ability to control for confounding, there is a strong interest in using large health care databases to study vaccine use and the outcomes of vaccination by projects such as the Vaccine Safety Datalink [8], Post-Licensure Rapid Immunization Safety Monitoring programme [9] and ADVANCE (http://www.advance-vaccines.eu/). Indeed, the size of observational databases allows for the study of rare events and, as they are embedded within clinical practice, they offer the potential to study real-world vaccine effects relatively efficiently from both cost and time perspectives.

When conducting VE studies it is important to quantify the potential impact of misclassification on the VE estimates in order to assess study feasibility, optimize study design and possibly, the need to correct for misclassification. In earlier work, the impact of non-differential disease misclassification on influenza VE has been quantified for cohort, case-control and test-negative designs based on mathematical derivations [10] and using simulation studies [10, 11]. We extended the simulation study by Orenstein [10] and Jackson [10, 11], to account for both disease- and exposure misclassification and allow for both differential and non-differential misclassification. Furthermore, as we show that the impact of misclassification on the estimated VE depends both on the epidemiology of the vaccine preventable disease and the expected vaccination coverage, we developed a web-application allowing to run simulations with user-defined parameters. We illustrate the impact of misclassification on VE estimates using two examples with clearly different disease attack rates and expected vaccination coverage; a) childhood pertussis and b) pediatric seasonal influenza VE estimations.

This work was carried out under the auspices of the ‘Accelerated development of vaccine benefit-risk collaboration in Europe’ (ADVANCE) project, launched in 2013, funded by the Innovative Medicines Initiative (IMI). The aim of ADVANCE is to help health professionals, regulatory agencies, public health institutions, vaccine manufacturers, and the general public make well-informed and timely decisions on benefits and risks of marketed vaccines by establishing a framework and toolbox to enable rapid delivery of reliable data on vaccine benefits and risks.

## Methods

In this section, we first present analytical derivations illustrating the impact of misclassification on VE estimates at population level—hence ignoring estimation error—when considering misclassification in its simplest form, being single source non-differential misclassification. Although estimation error is ignored, such analytical derivations provide meaningful insights. However, the derivations become tedious in situations where misclassification is more complex, especially when considering the joint impact of disease and exposure misclassification. Therefore, we also assess through simulation the impact of differential and non-differential disease and exposure misclassification when estimating VE using cohort, case-control, test-negative case-control and case-cohort (screening method) designs. These designs are used to estimate VE, with the classical cohort and case-control designs being probably the most commonly used ones [12]. The test-negative case-control design is popular for estimating VE of vaccines for influenza and rotavirus [13]. In the test-negative design, the study population are patients who are seeking medical care for a defined clinical condition (e.g. acute respiratory illness) and are tested for a specific viral infection (e.g. influenza). Then, patients testing positive are the cases and patients testing negative are the controls. Finally, the case-cohort or screening method uses data on the exposure prevalence in cases and compares this to the exposure prevalence from an external coverage cohort, from which the cases originate [14].

### Notation

First, let *π*_*VPD*.0_ be the unobserved ‘true’ risk of disease due to the pathogen targeted by the vaccine (vaccine preventable disease, VPD) in unvaccinated subjects, *π*_*Other*_ the corresponding risk of similar disease due to other pathogens than those targeted by the vaccine, and let *γ* be the ‘true’ vaccination coverage. Vaccination affects the VPD risk, with the risk among the vaccinated *π*_*VPD*.1_ = (1 − *VE*)*π*_*VPD*.0_, but does not affect the other disease risk. Furthermore, let *p*_0_ be the observed disease prevalence among the subjects indicated as unvaccinated and *p*_1_ the observed prevalence among the subjects indicated as vaccinated. Finally, let SE_d_ be the disease sensitivity (probability of being indicated as diseased if truly diseased) and SP_d_ the disease specificity (probability of being indicated as not diseased if truly not diseased) of the case definition. Similarly let SE_e_ be the exposure sensitivity (probability of being indicated as exposed if truly exposed) and SP_e_ the exposure specificity (probability of being indicated as unexposed if truly unexposed) of the exposure ascertainment definition. In the case of differential misclassification, the disease misclassification parameters depend on exposure status and vice versa, yielding four disease misclassification parameters; SE_d,E = 0_, SE_d,E = 1_, SP_d,E = 0_, SP_d,E = 1_ (with E = 0 indicating unvaccinated subjects and E = 1 vaccinated subjects) and four exposure misclassification parameters; SE_e,D = 0_, SE_e,D = 1_, SP_e,D = 0_, SP_e,D = 1_ (with D = 0 indicating not diseased subjects and D = 1 diseased subjects).

### Impact of misclassification at population-level

#### Non-differential disease misclassification

Given the simplifying assumptions of no exposure misclassification and no co-infection between the VPD and the similar disease due to other pathogens, the observed disease risk among the unvaccinated is the sum of the probability of having the VPD and being correctly indicated as such (true positive for disease) and the probability of having the non-VPD and being incorrectly indicated as having the VPD (false positive for disease) or

$$p_{0}={SE}_{d}\;\pi_{VPD.0}+(1-{SP}_{d})\pi_{Other}.$$ (1)

Similarly, for the vaccinated, the observed disease risk equals

$$p_{1}={SE}_{d}\;\pi_{VPD.1}+(1-{SP}_{d})\pi_{Other,}$$ (2)

with *π*_*VPD*.1_ = (1 − *VE*)*π*_*VPD*.0_.

In line with Orenstein [10] and analogous to the statistical definition of bias, we define the population-level bias as the difference in VE for a population with and without misclassification or

$$\Delta =(1-\frac{p_{1}}{p_{0}})-(1-\frac{\pi_{VPD.1}}{\pi_{VPD.0}})=\frac{\pi_{VPD.1}}{\pi_{VPD.0}}-\frac{{SE}_{d}\;\pi_{VPD.1}+(1-{SP}_{d})\pi_{Other}}{{SE}_{d}\;\pi_{VPD.0}+(1-{SP}_{d})\pi_{Other}}.$$ (3)

This expression can be rewritten as

$$\Delta =\frac{(\pi_{VPD.1}-\pi_{VPD.0})(1-{SP}_{d})\pi_{Other}}{\pi_{VPD.0}({SE}_{d}\;\pi_{VPD.0}+(1-{SP}_{d})\pi_{Other})},$$ (4)

showing that the bias equals zero if the disease specificity equals one, and this irrespective of the disease sensitivity.

Now, solving (1) for *π*_*VPD*.0_ and (2) for *π*_*VPD*.1_, we have

$$\pi_{VPD.0}=\left(p_{0}-(1-{SP}_{d})\pi_{Other}\right)/{SE}_{d}.$$ (5)

$$\pi_{VPD.1}=\left(p_{1}-(1-{SP}_{d})\pi_{Other}\right)/{SE}_{d},$$ (6)

based on which, and given accurate estimates of disease misclassification parameters, an estimate of the ‘true’ VE corrected for disease misclassification can be obtained as

$${VE}_{\pi }=1-\frac{p_{1}-(1-{SP}_{d})\pi_{Other}}{p_{0}-(1-{SP}_{d})\pi_{Other}}.$$ (7)

Interestingly, the correction equation requires an estimate of disease specificity but not of disease sensitivity. Obviously, the latter only holds if the disease misclassification is non-differential by vaccination status.

#### Non-differential exposure misclassification

Given the simplifying assumption of no disease misclassification the disease prevalence among subjects indicated as unvaccinated is the sum of the probability of having the VPD and being incorrectly indicated as unvaccinated (false negative for vaccination), and the probability of having the VPD and being correctly indicated as unvaccinated (true negative for vaccination) or

$$p_{0}={(1-SE}_{e})\;\gamma \;\pi_{VPD.1}+{SP}_{e}(1-\gamma )\pi_{VPD.0},$$ (8)

with true vaccination coverage *γ*. Similarly, the true positives and false positives for vaccination determine the disease risk among the subjects indicated as vaccinated or

$$p_{1}={SE}_{e}\;\gamma \;\pi_{VPD.1}+(1-{SP}_{e})(1-\gamma )\;\pi_{VPD.0}.$$ (9)

The population-level bias due to exposure misclassification is now defined as

$$\Delta =(1-\frac{p_{1}}{p_{0}})-(1-\frac{\pi_{VPD.1}}{\pi_{VPD.0}})=\frac{\pi_{VPD.1}}{\pi_{VPD.0}}-\frac{{SE}_{e}\;\gamma \;\pi_{VPD.1}+(1-{SP}_{e})(1-\gamma )\;\pi_{VPD.0}}{{(1-SE}_{e})\;\gamma \;\pi_{VPD.1}+{SP}_{e}(1-\gamma )\pi_{VPD.0}}.$$ (10)

This expression shows that the impact of sensitivity will be largest when coverage is high whereas the impact of specificity will be largest when coverage is low.

Solving (8) and (9) for *π*_*VPD*.0_ and for *π*_*VPD*.1_, we obtain

$$\pi_{VPD.0}=\left(p_{0}{SE}_{e}-p_{1}(1-{SE}_{e})\right)/(\left(1-\gamma )({SE}_{e}+{SP}_{e}-1\right)),$$ (11)

$$\pi_{VPD.1}=\left(p_{1}{SP}_{e}-p_{0}(1-{SP}_{e})\right)/\left(\gamma ({SP}_{e}+{SE}_{e}-1)\right).$$ (12)

Then, an expression of the ‘true’ *VE* corrected for exposure misclassification corresponds to

$${VE}_{\pi }=1-(\frac{1-\gamma }{\gamma })\frac{p_{1}{SP}_{e}-p_{0}(1-{SP}_{e})}{p_{0}{SE}_{e}-p_{1}(1-{SE}_{e})}.$$ (13)

This correction equation depends—next to the observed disease risks—on both exposure sensitivity and specificity as well as on the ‘true’ vaccination coverage.

### Simulation tool

Similar to Jackson [11], we simulate populations at risk for two outcomes; the VPD and a comparable outcome due to infection with one or more pathogen(s) not targeted by the respective vaccination. We assume that a number of subjects are vaccinated with coverage *γ*. Unvaccinated subjects could develop the VPD (only once) with a risk equal to *π*_*VPD*.0_ and the health outcome due to infection with other pathogens (only once) with a risk equal to *π*_*other*_. For vaccinated subjects, the risk of developing the VPD is reduced to *π*_*VPD*.1_ = (1 − *VE*)*π*_*VPD*.0_, whereas the risk due to other pathogens is unaffected by vaccination. We furthermore assume that the risks of developing both outcomes are independent. After having allocated the ‘true’ disease- and exposure status, we randomly allow these events to be misclassified. In particular, for the disease events, diseased cases are misclassified as not diseased with a probability of 1 − *SE*_*d*_ and not diseased cases are misclassified as diseased with a probability of 1 − *SP*_*d*_. The same holds for the exposure events, but using the exposure sensitivity *SE*_*e*_ and specificity *SP*_*e*_ parameters to simulate misclassification. In the case of differential misclassification, the disease misclassification parameters depend on exposure status and vice versa, yielding eight misclassification parameters in total; four disease misclassification parameters; SE_d,E = 0_, SE_d,E = 1_, SP_d,E = 0_, SP_d,E = 1_ and four exposure misclassification parameters; SE_e,D = 0_, SE_e,D = 1_, SP_e,D = 0_, SP_e,D = 1._

Then, for a given parameter setting, a large number of simulated populations (*k* = 1,2,…*K*) of a predefined population size *N* are generated. Based on the observed exposure and disease statuses in each population *k*, VE is estimated using the cohort, case-control, test-negative case-control and case-coverage designs, using case-cohort sampling as recommended in [10, 11] for the case-control designs (Table 1). Then, these estimates are compared with the true VE used to generate the simulated populations. The biases are compared graphically.

**Table 1:** Estimation of vaccine effectiveness (VE) for the cohort, case-control, test- negative case-control and case-coverage (screening method) design.

	Cohort	Case-control	Test-negative case-control	Screening method
For each simulated population	We calculate the VPD risk in the vaccinated vs in the unvaccinated.	We identify cases of VPD and sample controls from the full population at risk (case-cohort sampling); and for these two groups compare the odds of exposure as an odds ratio. We used case-cohort sampling as it was recommended in [10].	Here, the cases are the outcome events due to the VPD pathogen (test-positives) and the controls are the outcome events due to other pathogens (test-negatives).	We use only the exposure statuses of the observed cases and compare the odds of exposure in these cases with the odds of exposure in the external coverage cohort.
Estimate VE as	$\begin{array}{ll} {\hat{VE}}_{Co} & =1-{\hat{RR}}_{Co}\\ & =1-\frac{\hat{p_{v}}}{\hat{p_{u}}}, \end{array}$ with ${\hat{RR}}_{Co}$ the estimated ratio of the VPD risk in the vaccinated vs. unvaccinated; and estimated risks $\hat{p_{v}}$ and $\hat{p_{u}}$ based on observed proportions of VPD in the vaccinated and unvaccinated respectively.	$\begin{array}{ll} {\hat{VE}}_{\;CC} & =1-{\hat{OR}}_{CC}\\ & =1-\frac{\hat{p_{d}}/\left(1-\hat{p_{d}}\right)}{\hat{p_{n}}/\left(1-\hat{p_{n}}\right)}, \end{array}$ with ${\hat{OR}}_{CC}$ the estimated ratio of odds of exposure in cases vs. controls, which is equivalent to the odds of VPD in the vaccinated versus unvaccinated; and $\hat{p_{d}}$ and $\hat{p_{n}}$ being the observed proportions of exposure in the cases and controls respectively.	$\begin{array}{ll} {\hat{VE}}_{\;TN} & =1-{\hat{OR}}_{TN}\\ & =1-\frac{\hat{p_{tp}}/\left(1-\hat{p_{tp}}\right)}{\hat{p_{tn}}/\left(1-\hat{p_{tn}}\right)}, \end{array}$ with ${\hat{OR}}_{TN}$ the estimated ratio of the odds of exposure in the cases versus controls; $\hat{p_{tp}}$ and $\hat{p_{tn}}$ observed proportions of exposure in test-postitive and test-negative individuals respectively.	$\begin{array}{ll} {\hat{VE}}_{\;SCREEN} & =1-{\hat{OR}}_{SCREEN}\\ & =1-\frac{\hat{p_{d}}/\left(1-\hat{p_{d}}\right)}{\hat{X}/\left(1-\hat{X}\right)}, \end{array}$ with ${\hat{OR}}_{SCREEN}$ being the estimated ratio of the odds of exposure in the cases vs the odds of exposure in the external coverage cohort; $\hat{p_{d}}$ is as defined for the case-control design and $\hat{X}$ an estimate of the vaccine coverage for the external coverage cohort. For the simulation model, $\hat{X}$ is estimated as the proportion of individuals with observed exposures, assuming same levels of misclassification in the external coverage cohort as in the cases.

The simulation model is developed using R 3.3.1[15]. To allow modifying the simulations for other parameter settings/diseases while maximizing user-friendliness, we have encapsulated the source code of the simulation model in a web application created using the Shiny package [16]. Through the web application, the user can set all the necessary input parameters and the output files can be downloaded. The application can be found at the ADVANCE website (http://www.advance-vaccines.eu/) or at http://apps.p-95.com/VEMisclassification/.

### Scenarios

#### General settings

In this paper, we present two specific vaccination scenarios, pediatric seasonal influenza and childhood pertussis vaccination. For each subsequent simulation scenario, we set K = 1000 and N = 50 000 whereas VE, vaccination coverage and the respective attack rates depend on the specific scenarios detailed below. We vary one-by-one the disease- or exposure misclassification from {0.50,0.60,…1} while fixing the remaining misclassification parameters to 1.

#### Pediatric seasonal influenza

For consistency with Orenstein [10] and Jackson [11], we assumed a 1-dose VE of 70%, an attack rate (AR) of influenza in the unvaccinated of 15% and an AR of influenza-like illness not caused by influenza of 30%. The pediatric seasonal influenza vaccination coverage was assumed to be 10%, in line with the coverage rates reported for the majority of European countries [17].

#### Pertussis primary series

We assumed a VE of 80%, derived as a conservative value from a Cochrane systematic review of vaccine efficacy estimates obtained in random clinical trials, which found the efficacy of acellular pertussis vaccines in pediatric primary series to range between 71% and 85% for a follow-up period ranging from 17 to 22 months after vaccination [18]. We furthermore assumed that the AR of pertussis in the unvaccinated was 15% [19] and the AR of the non-vaccine preventable pathogens was 10.5% [20]. For the vaccination coverage, we assumed a value of 95%, which reflects a coverage rate commonly reported for the pediatric primary series in high-income countries[21].

## Results

### Pediatric seasonal influenza

**Fig 1:**
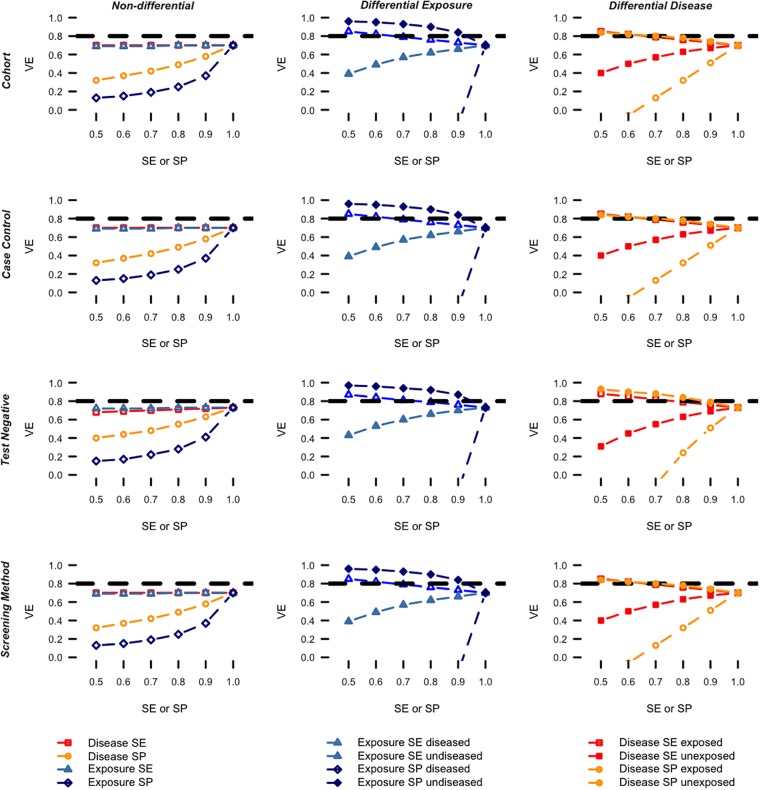
Influenza scenario: Vaccine effectiveness by design for varying levels of exposure- and disease misclassification while fixing the remaining parameters to 1. The dashed horizontal lines indicate the true VE used to simulate the data.

In the seasonal influenza scenario and assuming non-differential misclassification (Fig 1, left), the exposure specificity had the largest impact when fixing the remaining parameters to 1 followed by disease specificity and this across all designs. Indeed, the VE was most strongly underestimated when lowering the exposure specificity from 1 to 0.5. The underestimation in VE was still pronounced but less when lowering the disease specificity. Lowering the exposure sensitivity had a negligible impact on the VE whereas lowering the disease sensitivity had no impact when the remaining parameters were fixed to 1.

In case of differential exposure misclassification (Fig 1, middle), the bias could go in either direction, with the estimated VE showing very large deviations from the true VE. Across all designs, the exposure specificity for the diseased had the strongest impact among all four exposure misclassification parameters when fixing the remaining parameters to 1 and biases the VE estimates downwards. Also the exposure sensitivity for the undiseased yields a downwards bias. Lowering the exposure sensitivity for the diseased and the exposure specificity for the undiseased both show a slightly upwards bias.

In case of differential disease misclassification (Fig 1, right), the bias could go in either direction as well. Across all designs, the disease specificity for the exposed had the largest (downwards biasing) impact among the four disease misclassification parameters when fixing the remaining parameters to 1 followed by the disease sensitivity in the unexposed. The disease sensitivity for the exposed and the disease specificity for the unexposed are both associated with a slightly upwards bias. The test negative design performs worse than the other designs, particularly for low levels of disease specificity in the exposed.

### Pertussis primary series

**Fig 2:**
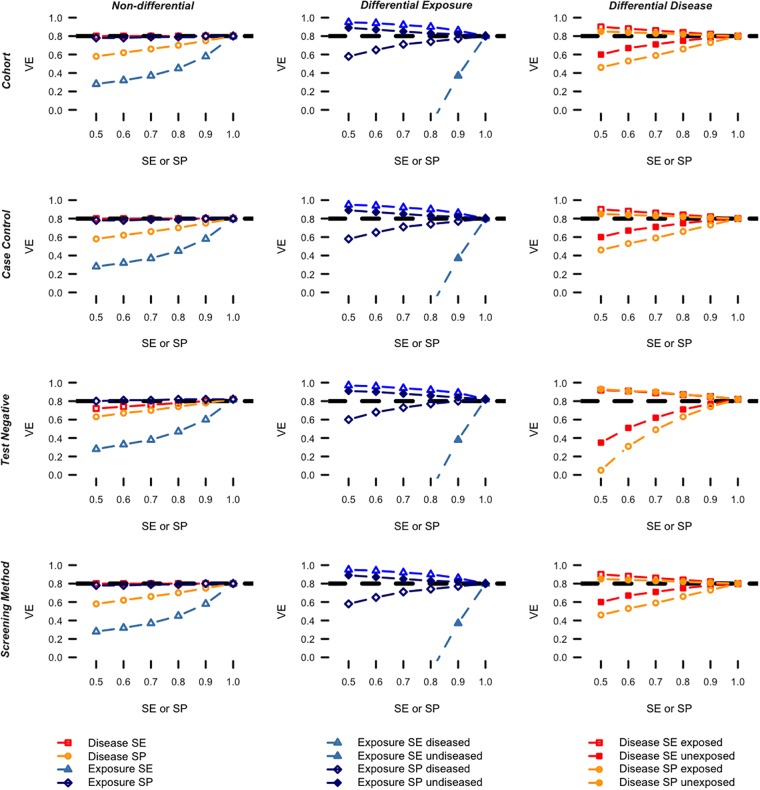
Pertussis scenario: Vaccine effectiveness by design for varying levels of exposure- and disease misclassification while fixing the remaining parameters to 1. The dashed horizontal lines indicate the true VE used to simulate the data.

In the pertussis scenario and assuming non-differential misclassification (Fig 2, left), the exposure sensitivity had the largest impact when fixing the remaining parameters to 1 followed by disease specificity. In case of differential exposure misclassification (Fig 2, middle), the exposure sensitivity for the un-diseased had the strongest impact among all four exposure misclassification parameters and biased the VE estimates downwards. Finally, in case of differential disease misclassification (Fig 2, right), the disease specificity for the exposed had the largest impact among the four disease misclassification parameters. The impact of the misclassification parameters was comparable across designs. As with pediatric influenza, the bias due to differential misclassification could go in either direction and lead to very large deviations from the true VE. Again, misclassification more strongly affects VE estimates from test-negative designs.

## Discussion

The development of the simulation tool has presented an opportunity to explore the interplay of disease- and exposure misclassification in VE estimations from different study designs. In this study, we explored the single impact of non-differential and differential disease- and exposure misclassification on childhood seasonal influenza and pertussis VE estimation. Depending on the scenario, the misclassification parameters had differing impacts. Decreased exposure specificity (poorer identification of non-vaccinees) had greatest impact for influenza VE estimation. Conversely decreased exposure sensitivity (poorer identification of vaccinees) had greatest impact for Pertussis VE estimation. These different impacts correspond to the respectively low and high vaccine coverage in the two scenarios, which is also supported by the analytical derivation (10) in Section 2.2. Similar observations were made regarding the impact of the exposure prevalence on the predictive values of the exposure assessment. Indeed, in low prevalence settings, the exposure specificity has the greatest impact with the lower the specificity the lower the positive predictive value. Conversely, in high prevalence settings, the exposure sensitivity has the greatest impact with the lower the sensitivity the lower the negative predictive value. Finally, it is interesting to note that, for the influenza and pertussis scenarios investigated, we found exposure misclassification to have a larger impact compared to disease misclassification whereas previous research focused on disease misclassification only.

The impact of the misclassification parameters was found to be more noticeable than that of the different study designs, with the different study designs performing similarly when misclassification is limited. Jackson [11] found earlier that VE estimates from test-negative case-control designs are more biased than those from classical cohort and case-control designs in case of substantial non-differential disease misclassification. We were able to replicate these results, and also found that the test-negative design performs particularly worse in case of substantial differential disease misclassification, with strong downward biases for low levels of disease specificity in the exposed. The worse performance of the test-negative design can be intuitively explained by comparing the case-control and test-negative design. In case-control designs, the false positives originate from the entire population of subjects free of the VPD with the false positive risk equal to the product of non-VPD risk and 1 minus the disease specificity. On the other hand, in test-negative designs, the false positives originate from the population of test-negatives with the false positive risk equal to 1 minus the disease specificity. Hence, the relative number of perturbations due to falsely classifying controls as cases is much smaller for the classical case-control design compared to the test-negative design.

Although the test-negative design is more sensitive to disease misclassification compared to other designs, its performance remains good when misclassification is limited. Next to misclassification, other sources of bias such a confounding and selection bias should be considered when selecting an appropriate study design. For instance, observational studies on influenza VE might be strongly confounded by differences in healthcare seeking behavior between vaccinated and unvaccinated persons, therefore the test-negative design might still be the appropriate choice in this case [22].

The dependence of the impact of misclassification on the scenario urged us to develop a user-friendly simulation tool that can be modified by users to their own study scenario. The tool allows users to assess the single and joint impact of both differential and non-differential disease- and exposure misclassification on VE estimates from cohort, case-control, test-negative case-control and case-coverage studies. The simulation tool can be accessed through the ADVANCE website (http://www.advance-vaccines.eu/) or using http://apps.p-95.com/VEMisclassification/.

It is well-known that exposure- and disease misclassification might jeopardize the validity of VE studies and that such studies require careful design. The simulation tool might help researchers to anticipate at design stage the magnitude and direction of the bias when estimating VE based on potentially misclassified data. As such, this tool can guide the selection of the exposure- and disease definitions that will minimize bias due to misclassification. In addition, if the potential impact of misclassification is found to be unacceptable, several methods to adjust estimates for misclassification exist, although they are not yet commonly used in pharmacoepidemiology [23]. We provided the correction equations for VE estimates in case of non-differential single source (either exposure or disease) misclassification (Section 2.2). Other correction methods include amongst others probabilistic bias analyses [24, 25], Bayesian bias analyses [26–28], modified maximum likelihood methods [29] and imputation-like methods [30–33]. All these methods require assumptions on or estimates of the disease- and exposure misclassification parameters, which—if deemed required—can be obtained using internal or external validation studies.

Several limitations or areas of further development are worth considering. The simulation tool singles out the impact of disease- and exposure misclassification and ignores other sources of bias. Specifically, it is assumed that there is no confounding and no selection bias. In addition, the tool does not include dependent misclassification. For binary variables, misclassification is dependent when the probability of misclassification of one variable depends on the correctness of classification on the other variable [34]. Dependent measurement errors might arise, for example, if data on both exposure and outcome were obtained from medical records with data paucity for some but not all subjects. Furthermore, the tool assumes binary disease- and exposure variables, whereas particularly the exposure variable might be polytomous (no vaccination, partial or complete vaccination).

The results presented in this paper and the simulation tool may be useful to guide researchers to better design, conduct and interpret future VE studies when data are subject to misclassification. We advocate to use such a simulation tool and modify the parameters according to the study specifics since we have shown that the impact of misclassification strongly depends on the study scenario.
